# Mitochondrial Reactive Oxygen Species Mediate Activation of TRPV1 and Calcium Entry Following Peripheral Sensory Axotomy

**DOI:** 10.3389/fnmol.2022.852181

**Published:** 2022-03-18

**Authors:** Bradley Kievit, Aaron D. Johnstone, Julien Gibon, Philip A. Barker

**Affiliations:** ^1^Department of Biology, University of British Columbia Okanagan, Kelowna, BC, Canada; ^2^Department of Neurology and Neurosurgery, Montreal Neurological Institute, McGill University, Montreal, QC, Canada

**Keywords:** axotomy, TRPV1, mitochondria, ROS, degeneration

## Abstract

Axons that are physically separated from their soma activate a series of signaling events that results in axonal self-destruction. A critical element of this signaling pathway is an intra-axonal calcium rise that occurs just prior to axonal fragmentation. Previous studies have shown that preventing this calcium rise delays the onset of axon fragmentation, yet the ion channels responsible for the influx, and the mechanisms by which they are activated, are largely unknown. Axonal injury can be modeled *in vitro* by transecting murine dorsal root ganglia (DRG) sensory axons. We coupled transections with intra-axonal calcium imaging and found that Ca^2+^ influx is sharply reduced in axons lacking *trpv1* (for transient receptor potential cation channel vanilloid 1) and in axons treated with capsazepine (CPZ), a TRPV1 antagonist. Sensory neurons from *trpv1*^–/–^ mice were partially rescued from degeneration after transection, indicating that TRPV1 normally plays a pro-degenerative role after axonal injury. TRPV1 activity can be regulated by direct post-translational modification induced by reactive oxygen species (ROS). Here, we tested the hypothesis that mitochondrial ROS production induced by axotomy is required for TRPV1 activity and subsequent axonal degeneration. We found that reducing mitochondrial depolarization with NAD^+^ supplementation or scavenging ROS using NAC or MitoQ sharply attenuates TRPV1-dependent calcium influx induced by axotomy. This study shows that ROS-dependent TRPV1 activation is required for Ca^2+^ entry after axotomy.

## Introduction

Peripheral axons subjected to transection *in vivo* rapidly undergo an active degenerative program termed Wallerian degeneration. A hallmark event in this process, initially described over 40 years ago, is a rise in intra-axonal calcium (Schlaepfer and Bunge, 1973; George et al., 1995; Avery et al., 2012; Villegas et al., 2014), with an initial increase in calcium near the site of axotomy (Kerschensteiner et al., 2005; Breckwoldt et al., 2014) followed several hours later by an increase that occurs throughout the entire distal axon. This second rise in intra-axonal calcium precedes overt axonal degeneration (Adalbert et al., 2012; Vargas et al., 2015). The late phase of calcium rise has been proposed to rely on the influx of extracellular ions through membrane-bound voltage-gated channels (George et al., 1995; Knöferle et al., 2010) and the Na^+^/Ca^2+^ exchanger (NCX) (Lehning et al., 1996; Persson et al., 2013) as well as from intracellular sources.

The molecular mechanisms that mediate the late phase of calcium rise remain largely uncharacterized. One clue comes from studies on the Wallerian Degeneration Slow (WldS) protein, a chimeric protein composed of the NAD^+^-synthesizing enzyme nicotinamide mononucleotide acetyltransferase (NMNAT) and a fragment of ubiquitination factor UBE4B. This fusion protein not only protects axons from degeneration after injury *in vitro* and *in vivo* (Lunn et al., 1989; Coleman et al., 1998; Conforti et al., 2000; Mack et al., 2001; Wang et al., 2015) but also sharply reduces the later stage of axonal Ca^2+^ increase (Adalbert et al., 2012; Vargas et al., 2015). Axotomy triggers mitochondrial depolarization, opens the mitochondrial permeability transition pore, and results in mitochondrial ROS production and their accumulation in the axoplasm (Barrientos et al., 2011; Korge et al., 2011). Studies using mitochondrial poisons such as carbonyl cyanide m-chlorophenyl hydrazine (CCCP) have indicated that mitochondrial depolarization activates cell surface calcium channels but the signaling mechanisms that connect these events remain uncertain (Nesuashvili et al., 2013; Stanford and Taylor-Clark, 2018).

TRPV1 is a non-selective cation channel widely expressed in the peripheral and the central nervous systems (Cui et al., 2006). TRPV1 plays a critical role in nociception and can be activated by protons, heat above 43°C, oxidized lipid products, endogenous ligands such as anandamide and exogenous compounds such as capsaicin (Caterina et al., 1997). An essential feature of TRPV1 is that it is sensitized by ROS (Chuang and Lin, 2009; Keeble et al., 2009). This may be relevant in axonal degeneration because axotomy results in ROS production (O’Donnell et al., 2013) and ROS scavenging delays axonal degeneration after transection (Frati et al., 2017). Notably, TRPV1 is a target for treating some forms of neuropathy, and chronic TRPV1 activation can induce axon terminal ablation (Simone et al., 1998; Wang et al., 2017).

Mitochondrial potential collapse and increases in intracellular calcium levels have been causally linked in neuronal disease states (Maher and Schubert, 2000; Mattson, 2007; Court and Coleman, 2012; Wilson et al., 2016; Carrasco et al., 2018) but their mechanistic links remain obscure. Here, we examined the interplay of these after axotomy. Using a combination of pharmacological and genetic tools, we show that TRPV1 contributes to Ca^2+^ rise after axotomy. Activation of TRPV1 after axotomy is blocked by NAD^+^ and by ROS scavenging, suggesting that mitochondrial-derived ROS are required for TRPV1 activation in this setting. Consistent with this, we show that TRPV1 is activated in intact axons challenged with CCCP through a ROS-dependent mechanism. Taken together, these data demonstrate a crucial role of mitochondrial ROS-dependent activation of TRPV1 in the calcium entry that precedes overt degeneration after axotomy.

## Materials and Methods

### Animal Strains

Wildtype CD-1 and C57Bl/6J mice were purchased from Charles River laboratories. *Trpv1*^–/–^ C57Bl/6J mice carrying the *TRPV1*^TM 1*Jul*^ (Caterina et al., 1997) were purchased from Jackson Laboratories (United States). Animals were maintained in a 12 h light/dark cycle and had access to food, water, ad libitum. All animal procedures and experiments were approved by the UBC Animal Care Committee. Efforts were made to reduce animal handling and use.

### Antibodies and Reagents

Antibodies directed against β-III tubulin (Tuj1, 1:10,000 for immunofluorescence, MAB5564) were purchased from Millipore (Canada). The TRPV1 inhibitor capsazepine (CPZ) was obtained from Tocris Biosciences and was used at a final concentration of 10 μM. The TRPV1 agonist capsaicin (Sigma Aldrich, Canada) was used at 1 μM. The L-type channel inhibitor nifedipine was purchased from Sigma Aldrich used at 10 μM. NAD^+^ (Sigma Aldrich, Canada) and N-acetyl-L-cysteine (NAC, Sigma Aldrich, Canada) were used at final concentrations of 5 mM and 20 mM, respectively. EGTA (VWR, Canada) was used at 6 mM. The mitochondrial ROS scavenger MitoQ (a generous gift of Dr. Michael Murphy) was used at 1 μM (Kelso et al., 2001). CCCP (Sigma Aldrich, Canada) was used at a final concentration of 50 μM.

### Dorsal Root Ganglion Culture and Treatments

Dorsal root ganglia (DRG) explants were prepared from E13.5 mouse embryos and grown on cell culture filter inserts (1 μm pore size; BD-Falcon, Canada), 4-chamber glass bottom dishes (Cellvis, United States) or 6-well plastic culture plates (VWR, Canada) that had been coated sequentially with poly-D-lysine (1 mg/ml; Sigma-Aldrich, Canada), laminin (10 μg/ml; Sigma-Aldrich, Canada), and collagen (0.1 mg/ml, PureCol; Advance BioMatrix, United States). Culture media consisted of Neurobasal media (Invitrogen, Canada) supplemented with 2% B-27 (Invitrogen, United States), 1% L-glutamine (Wisent, Canada), 1% penicillin/streptomycin (Wisent, Canada), 20 μM 5-fluoro-2’-deoxyuridine (Sigma-Aldrich, Canada) and 12.5 ng/ml NGF (Alomone, Israel). Axotomy was achieved *in vitro* on filter inserts by scraping the top of the filters and maintaining axons at 37°C for the time periods as indicated in the text. It is noteworthy that our preparations involve severing afferents when DRGs are removed from the animal and involve later severing axons *in vitro*. Therefore, DRG sensory neurons in our preparation are axotomized twice prior to analyses. For fluo-4 axotomy experiments, DRG maintained on glass plates were transected under a dissecting microscope (20×) and using a scalpel blade. DRG were then maintained at 37°C for the time periods indicated.

### Axonal Ca^2+^ and Mitochondrial Depolarization Analysis

Fluo-4-AM (1 μM) or the mitochondrial dye tetramethylrhodamine (TMRE, 100 nM) were dissolved in DMSO (0.1% v/v), and then added to culture plates for 15 min at 37°C. For Calcein-AM experiments, axons were incubated with the dye for 1 h. Axons were washed in HBSS and then maintained in HBSS supplemented with 12.5 ng/ml NGF and 2 mM CaCl_2_ during imaging. Imaging was performed using a Zeiss AxioObserver Z1 and Zen software.

For endpoint Ca^2+^ imaging, axotomized DRG cultures grown on glass plates were incubated with the Ca^2+^-sensor dye fluo-4-AM and imaged with a 40× objective 3 h after axotomy. For endpoint TMRE imaging, axotomized DRG cultures grown on glass plates were incubated with calcein-AM and TMRE and imaged with a 40× objective 3.5 h after axotomy. To image axonal Ca^2+^ levels before and after mitochondrial depolarization, DRG cultures were loaded with fluo-4 and with TMRE and images were captured every 5 s. After a baseline was established (*t* = 0–100 s), CCCP was added and images were collected for an additional 400 s.

### Axonal Degeneration Analysis on Filter Inserts or Culture Plates

DRGs axonal degeneration analyses were performed as previously described (Unsain et al., 2014). Briefly, DRGs grown on filters or plastic culture plates were fixed with 4% paraformaldehyde for 20 min at room temperature and then incubated in blocking solution (TBS-T, 5% skim milk, and 0.3% Triton X-100) for 1 h. DRGs were then incubated overnight with antibodies against β-III tubulin, diluted 1:10,000 in blocking solution. Plates or filters were then incubated with Alexa488-conjugated goat anti-mouse secondary antibodies (Jackson Laboratory, United States) for 2 h at room temperature. The filters were removed from the insert, placed in fluorescent mounting medium (Fluoroshield, Sigma Aldrich, Canada), and mounted on Superfrost Plus slides (Fisher Scientific, Canada) then sealed with microscope cover glass (Fisher Scientific, Canada). Imaging was performed using a Zeiss AxioObserverZ1 inverted epifluorescence microscope at 5× magnification with an automated, motorized stage. Tiled images were stitched automatically with Zen 2 software to produce images of the entire culture plate or filter.

### Imaging and Statistical Analyses

Fluo-4-AM intensities in endpoint and live axonal Ca^2+^ experiments were quantified using ImageJ software (FIJI build, National Institute of Health, Bethesda, MD, United States). Background intensity was calculated as the mean intensity of 4 selected axon-free regions of interest (ROIs) within a given image. A threshold was established based on individual pixel intensities to separate axonal area from background. The mean intensity of pixels within the binary mask generated from the threshold image was recorded and the background intensity was subtracted. For endpoint Ca^2+^ experiments, plotted fluorescence values represent the average of at least four fields taken from the same explant, normalized to the mean intensity of intact untreated controls. For Ca^2+^ imaging of live axons, the intensity of the time course was normalized to the intensity level of the first image and a single replicate represents the mean of three time courses done in a single experiment day for each condition.

Axonal density in axonal degeneration experiments was quantified by Axoquant 2.0 as previously described (Johnstone et al., 2018). Mitochondria polarization status was quantified using a threshold mask that created a binary image displaying cross-sectional axon area and cross-sectional mitochondria area based on calcein and TMRE-stained images. Mitochondria polarization status was calculated as the area of TMRE signal (total mitochondrial area) divided by the area of calcein signal (total axon area). Each replicate represents an average of at least four images of the same DRG explant, normalized to images of control DRG explants.

Each replicate (= each N) represents a distinct embryo. All statistical analyses were performed using a two-way ANOVA. Significance was established at *P* < 0.05. Data are presented as boxplots indicating median, 25%, 75% percentiles as well as minimum/maximum data points. Axon degeneration data are displayed as means ± standard error of the mean (S.E.M.).

## Results

### Late-Stage Ca^2+^ Influx Is Reduced by TRPV1 Inhibition in Axons After Transection

**FIGURE 1:**
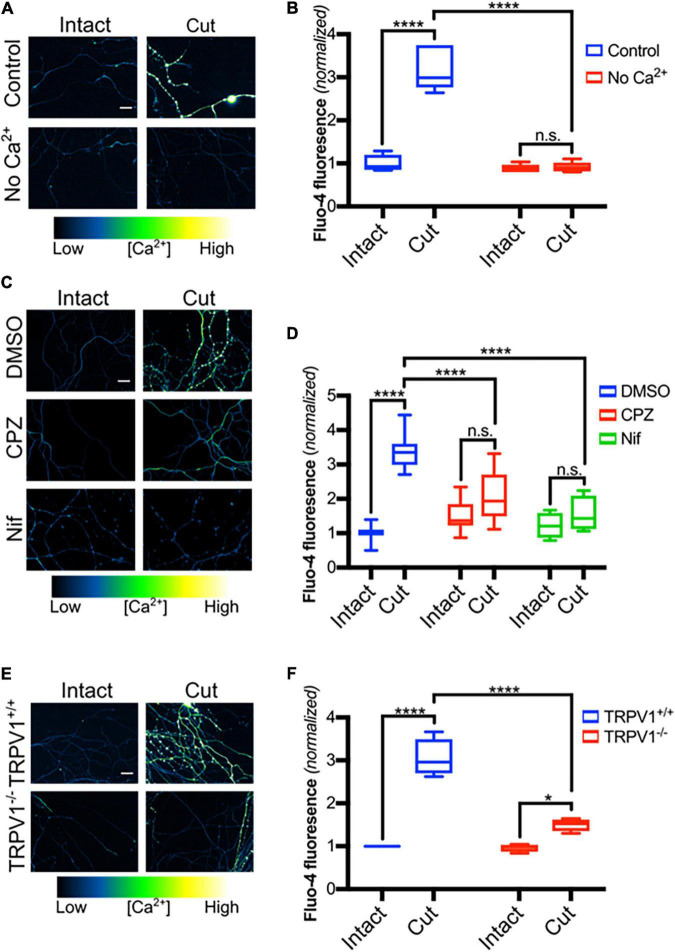
TRPV1 inhibition reduces Ca^2+^ influx in transected axons. **(A)** Micrographs of intact or cut DRG axons in the presence or absence of Ca^2+^ (No Ca^2+^), loaded with Fluo-4-AM (scale bar indicates 25 μm) and imaged 3 h after axotomy. **(B)** Fluo-4 fluorescence intensity, normalized to fluorescence intensity of intact controls (*n* = 6 for each group). Boxplots show minimum, 1st quartile, median, 3rd quartile, maximum. Statistical significance was assessed using a two-factor ANOVA followed by Tukey’s *post hoc* comparison. **(C)** Intact or cut axons in the presence or absence of CPZ (10 μM – 30 min pre-incubation), nifedipine (Nif, 10 μM -30 min pre-incubation) or DMSO (0.1% v/v- – 30 min pre-incubation), loaded with Fluo-4-AM (scale bar indicates 25 μm) and imaged 3 h after axotomy. **(D)** Quantified Fluo-4 fluorescence intensity normalized to fluorescence intensity of intact DMSO controls [*n* = 10 (DMSO), 8 (CPZ), 6 (Nif)]. Boxplots are minimum, 1st quartile, median, 3rd quartile, maximum. Statistical significance was assessed using a two-factor ANOVA followed by Tukey’s *post hoc* comparison. **(E)** Micrographs of *trpv1*^+/+^ and *trpv1*^– /–^ axons that were loaded with Fluo-4-AM and left intact or axotomized (scale bar indicates 25 μm). **(F)** Fluo-4 fluorescence intensity normalized to fluorescence intensity of the intact *Trpv1*^+/+^ group (*n* = 4 for each group). Boxplots are minimum, 1st quartile, median, 3rd quartile, maximum. Statistical significance was assessed using a two-factor ANOVA followed by Tukey’s *post hoc* comparison. Values are mean ± SEM. Statistical significance was assessed using a two-factor ANOVA followed by Dunnett’s test relative to Ax + DMSO **(F)**, *p < 0.05, ****p < 0.0001.

Axon transection causes an immediate rise of calcium levels within the distal axon that is likely due to passive influx through the lesion site (Breckwoldt et al., 2014; Vargas et al., 2015). Intra-axonal calcium levels quickly fall back to baseline but then after a delay of hours, calcium levels within distal transected segments again increase. Previous studies have suggested that this reflects influx of extracellular calcium *via* L-type calcium channels and that this entry plays a critical role driving axonal destruction (Schlaepfer and Bunge, 1973; George et al., 1995; Adalbert et al., 2012; Vargas et al., 2015). To begin to assess molecular components of this late phase of calcium influx, we established a DRG transection model in which sensory axons were blade transected 100 μM from the soma. Transected DRGs were placed in Ca^2+^-free media or in media containing 2 mM Ca^2+^ for 3 h and then subjected to Fluo-4 imaging (Figure 1). As expected, transected DRG axons maintained in extracellular calcium show a large increase in intra-axonal Ca^2+^ after lesion whereas those maintained in calcium-free medium lack this response (Figures 1A,B). Interestingly, Villegas et al. (2014) have shown that release of Ca^2+^ from intracellular stores can occur later, at 12 h post-axotomy.

We recently showed that TRPV1 plays a critical role regulating calcium entry during developmental axonal degeneration (Johnstone et al., 2019) and hypothesized that TRPV1 also contributes to calcium entry after axonal transection. Because L-type calcium channels have previously been identified as a route of calcium entry after axotomy (George et al., 1995), we compared late phase Ca^2+^ entry in DRG axons exposed to nifedipine, which blocks L-type calcium channels, to axons exposed to capsazepine (CPZ), which blocks TRPV1. Figures 1C,D shows that both agents sharply reduce the Ca^2+^ influx that normally occurs after axotomy suggesting that both TRPV1 and L-type calcium channels play significant roles regulating calcium entry after DRG sensory neuron axotomy.

To confirm that TRPV1 is required for Ca^2+^ entry after axonal transection, DRGs derived from *trpv1* null mice were subjected to blade axotomy and imaged using Fluo-4. Figures 1E,F shows that calcium accumulation within transected axons was sharply reduced in mice lacking *trpv1*, confirming a key role for TRPV1 in the late phase of calcium entry after lesion.

**FIGURE 2:**
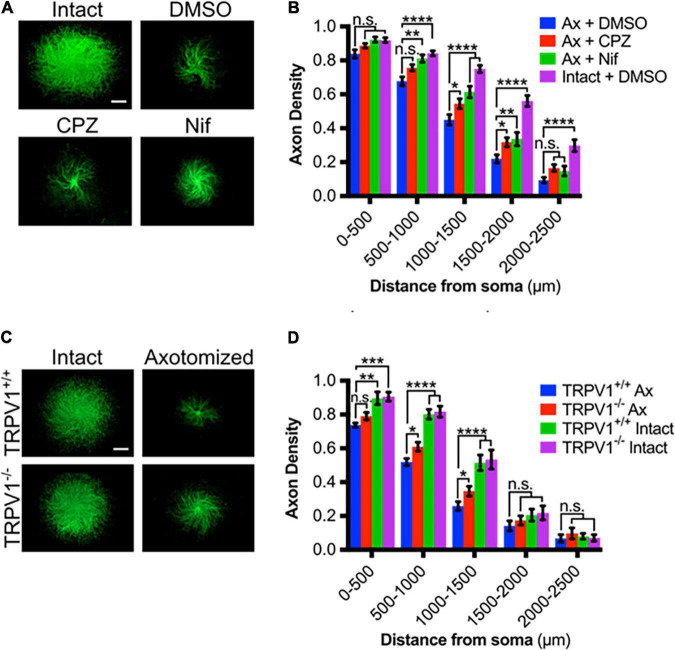
TRPV1 is required for Ca^2+^ influx in transected axons. **(A)** Intact or axotomized DRG axons in the absence or presence of CPZ (10 μM – 30 min pre-incubation), Nif (10 μM – 30 min pre-incubation), or DMSO (0.1% v/v – 30 min pre-incubation), immunostained for β-III tubulin (scale bar indicates 1,000 μm). **(B)** Axon density of intact or axotomized (Ax) DRGs in radial bins of increasing distance from the center [*n* = 17 (Ax + CPZ), *n* = 16 (Ax + Nif), *n* = 13 (Ax + DMSO)]. **(C)** Intact or axotomized *trpv1*^+/+^ and *trpv1*^– /–^ DRG axons immunostained for β-III tubulin (scale bar indicates 1,000 μm). **(D)** Axon density of intact or axotomized (Ax) DRGs in radial bins of increasing distance from the center [*n* = 6 (*trpv1*^+/+^ Ax and *trpv1*^– /–^ Ax), *n* = 3 (*trpv1*^+/+^ Intact and *trpv1*^–^*^/^*^–^ Intact)]. Values are mean ± SEM. Statistical significance was assessed using a two-factor ANOVA followed by Dunnett’s test relative to *trpv1*^+/+^ Ax **(D)**, *p < 0.05, **p < 0.01, ***p < 0.001, ****p < 0.0001.

We then asked if TRPV1 blockade reduces degeneration after axotomy. For this, we examined axotomy *in vitro* using DRG explants maintained on porous filters as previously described. DRG were established on the filters for 48 h and then cell bodies on the top surface were removed and axons remaining on the filter’s bottom surface were assessed for degeneration at several discrete distances from their origin [see section “Materials and Methods” as well as Unsain et al. (2014)]. We directly compared CPZ and nifedipine in this setting and found that each of these agents effectively reduces axonal degeneration 6 h after axotomy (Figures 2A,B). This indicates that Ca^2+^ entry *via* TRPV1 and L-type channels is required for complete DRG sensory axon degeneration after lesion.

The effect of *trpv1* gene deletion on DRG axonal degeneration was also assessed; Figures 2C,D shows that *trpv1*^–/–^ axons show a significant, albeit modest reduction in injury-induced axonal degeneration. We conclude that calcium entry *via* TRPV1 is required for a normal axonal injury response.

### Mitochondrial Reactive Oxygen Species Is Required Following Axotomy for Late-Stage Ca^2+^ Flux in Distal Axons

**FIGURE 3:**
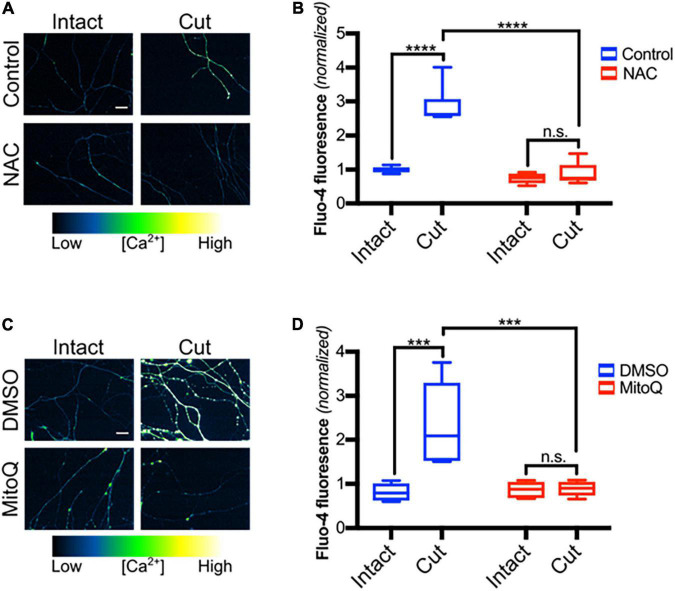
Mitochondrial reactive oxygen species are required for calcium influx after axotomy. **(A)** Transected and intact sensory axons in the presence of NAC (20 mM -30 min pre-incubation), loaded with Fluo-4-AM (scale bar indicates 25 μm) and imaged 3 h after axotomy. **(B)** Fluo-4-AM intensity within axons, normalized to fluorescence of intact control [*n* = 6 (control), 5 (NAC)]. Boxplots are minimum, 1st quartile, median, 3rd quartile, maximum. Statistical significance was assessed using a two-factor ANOVA followed by Tukey’s *post hoc* comparison. **(C)** Micrographs of intact or cut axons pretreated with DMSO (0.1% v/v -30 min pre-incubation) or MitoQ (1 μM -30 min pre-incubation), loaded with Fluo-4-AM (scale bar indicates 25 μm) and imaged 3 h after axotomy. **(D)** Fluo-4 fluorescence intensity normalized to fluorescence of intact control (*n* = 6 for all groups). Boxplots are minimum, 1st quartile, median, 3rd quartile, maximum. Statistical significance was assessed using a two-factor ANOVA followed by Tukey’s *post hoc* comparison, ***p < 0.001, ****p < 0.0001.

Because TRPV1 can be directly regulated by redox state and reactive oxygen species (ROS) have been implicated in post-axotomy events (Press and Milbrandt, 2008; Welin et al., 2009; O’Donnell et al., 2013; Wakatsuki et al., 2015), we hypothesized that ROS contributes to TRPV1 activation in transected axons. To address this, DRGs were pre-treated with NAC, a ROS scavenger, subjected to blade axotomy, and imaged for Ca^2+^ levels 3 h later (Figure 3A). Figures 3A,B show that NAC treatment suppressed the intra-axonal calcium increase that normally occurs after transection, indicating that ROS do indeed play a critical role regulating Ca^2+^ entry in this setting. Several studies have established that mitochondrial failure leads to increased ROS production within transected distal axons (Sievers et al., 2003; Avery et al., 2012; O’Donnell et al., 2013; Godzik and Coleman, 2015) and we hypothesized that mitochondrial-derived ROS may contribute to post-axotomy calcium influx. To address this, DRGs were pretreated with MitoQ, an anti-oxidant that concentrates ∼100-fold in mitochondria (Murphy and Smith, 2007), and axons were then subjected to axotomy. Figure 3C shows that the late-stage calcium influx that normally occurs 3 h after axotomy was abolished in DRGs exposed to MitoQ (Figures 3C,D) consistent with the hypothesis that mitochondrial ROS production is required for injury-induced calcium entry.

### NAD^+^ Supplementation Rescues Mitochondrial Depolarization and Blocks Calcium Influx

**FIGURE 4:**
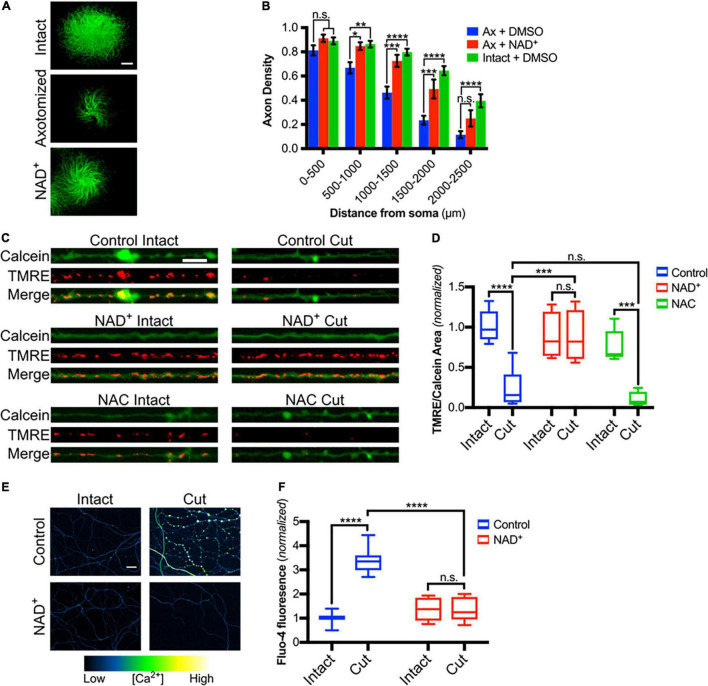
Mitochondrial depolarization induced by axotomy is dependent on loss of NAD^+^ but not on ROS accumulation. **(A)** Intact or cut DRG axons treated with NAD^+^ (5 mM-30 min treatment) and immunostained for β-III tubulin (scale bar indicates 1,000μm). **(B)** Axon density of intact or axotomized (Ax) DRGs in radial bins of increasing distance from the center (*n* = 7 for each group). Values are mean ± SEM. Statistical significance was assessed using a two-factor ANOVA followed by Dunnett’s test relative to Ax + DMSO. **(C)** Intact or cut axons treated with either NAD^+^ (5 mM) or NAC (20 mM), loaded with Calcein-AM and TMRE (scale bar indicates 10 μm) and imaged 3.5 h after axotomy. **(D)** Mitochondrial polarization readout calculated as TMRE-positive area divided by Calcein-positive area and normalized to intact control [*n* = 10 (control), 5 (NAC), 4 (NAD^+^)]. Boxplots are minimum, 1st quartile, median, 3rd quartile, maximum. Statistical significance was assessed using a two-factor ANOVA followed by Tukey’s *post hoc* comparison. **(E)** Micrographs of intact or cut axons treated with NAD^+^ (5 mM) and loaded with Fluo-4-AM (scale bar indicates 25 μm). **(F)** Fluo-4-AM intensity in axons, normalized to intensity of intact controls [*n* = 10 (control), 7 (NAD^+^)]. Boxplots are minimum, 1st quartile, median, 3rd quartile, maximum. Statistical significance was assessed using a two-factor ANOVA followed by Tukey’s *post hoc* comparison, *p < 0.05, **p < 0.01, ***p < 0.001, ****p < 0.0001.

Multiple studies have established the critical role of axonal NAD^+^ depletion in axonal degeneration (Wang et al., 2005; Avery et al., 2009; Gilley and Coleman, 2010; Sasaki and Milbrandt, 2010). To confirm that NAD^+^ rescues DRG sensory axons from axotomy-induced degeneration in our setting, DRGs were grown on porous membranes, pretreated with NAD^+^ for 30 min and then cell bodies were scraped from the top of the membrane. Axons remaining on the bottom of the filter were maintained in NAD^+^ for 6 h and then assessed for degeneration. As expected, NAD^+^ supplementation protects axons from post-transfection degeneration (Figures 4A,B). Taken together, these data are consistent with a model in which NAD^+^ depletion results in mitochondrial depolarization and ROS generation which in turn leads to redox modification of TRPV1 and calcium entry. To address this, we first examined mitochondrial potential in DRG axons treated with either NAD^+^ or NAC and then subjected to axotomy. Using TMRE fluorescence as a read-out for polarized mitochondria, we found that mitochondrial polarization is lost at 3.5 h after axotomy. Figures 4C,D show that this loss is blocked in axons treated with NAD^+^ but not in those treated with NAC. We then asked if blocking mitochondrial depolarization using NAD^+^ supplementation reduces post-axotomy calcium entry. For this, DRGs were pre-treated with NAD^+^ for 30 min, axons were severed and intra-axonal calcium levels were assessed 3 h later. Figures 4E,F shows that the late phase of intra-axonal calcium accumulation is lost in axons provided with NAD^+^ supplementation.

### Carbonyl Cyanide m-Chlorophenyl Hydrazine-Induced Axonal Degeneration Requires Reactive Oxygen Species Accumulation and TRPV1 Activation

**FIGURE 5:**
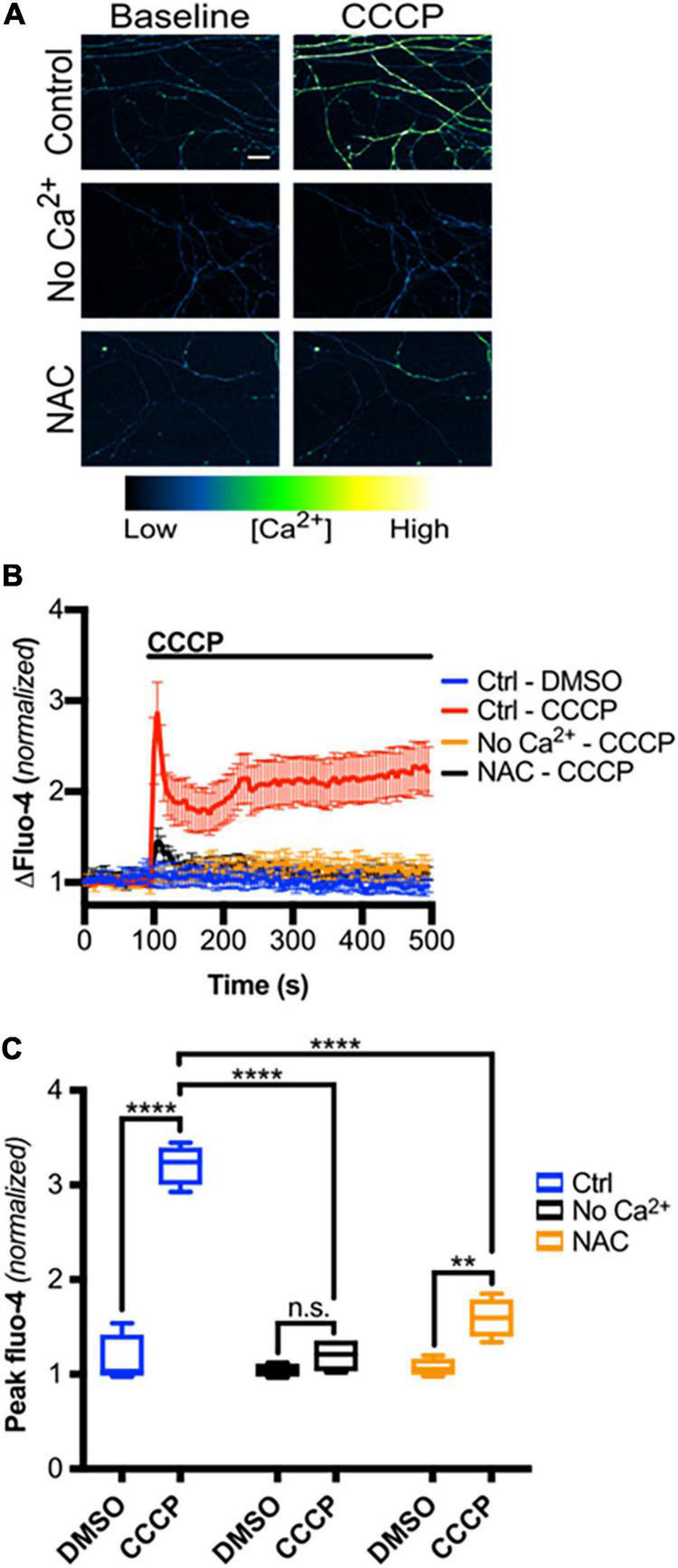
Mitochondrial depolarization in intact axons induces ROS-dependent calcium influx. **(A)** Micrographs of Fluo-4-AM-loaded axons, treated with Ca^2+^-free media or NAC (20 mM), before and immediately after CCCP treatment (50 μM) (scale bar indicates 25 μm). **(B)** Changes in Fluo-4 intensity before and after CCCP treatment, normalized to intensities at *t* = 0 s [*n* = 8 (Ctrl – DMSO, Ctrl – CCCP), 4 (No Ca^2+^ – CCCP, NAC – CCCP)]. Values are mean ± SEM. **(C)** Peak Fluo-4-AM intensities immediately following CCCP treatment (taken from panel **B**), normalized to intensity at *t* = 0 s. Boxplots are minimum, 1st quartile, median, 3rd quartile, maximum. Statistical significance was assessed using a two-factor ANOVA followed by Tukey’s *post hoc* comparison. **p < 0.01, ****p < 0.0001.

**FIGURE 6:**
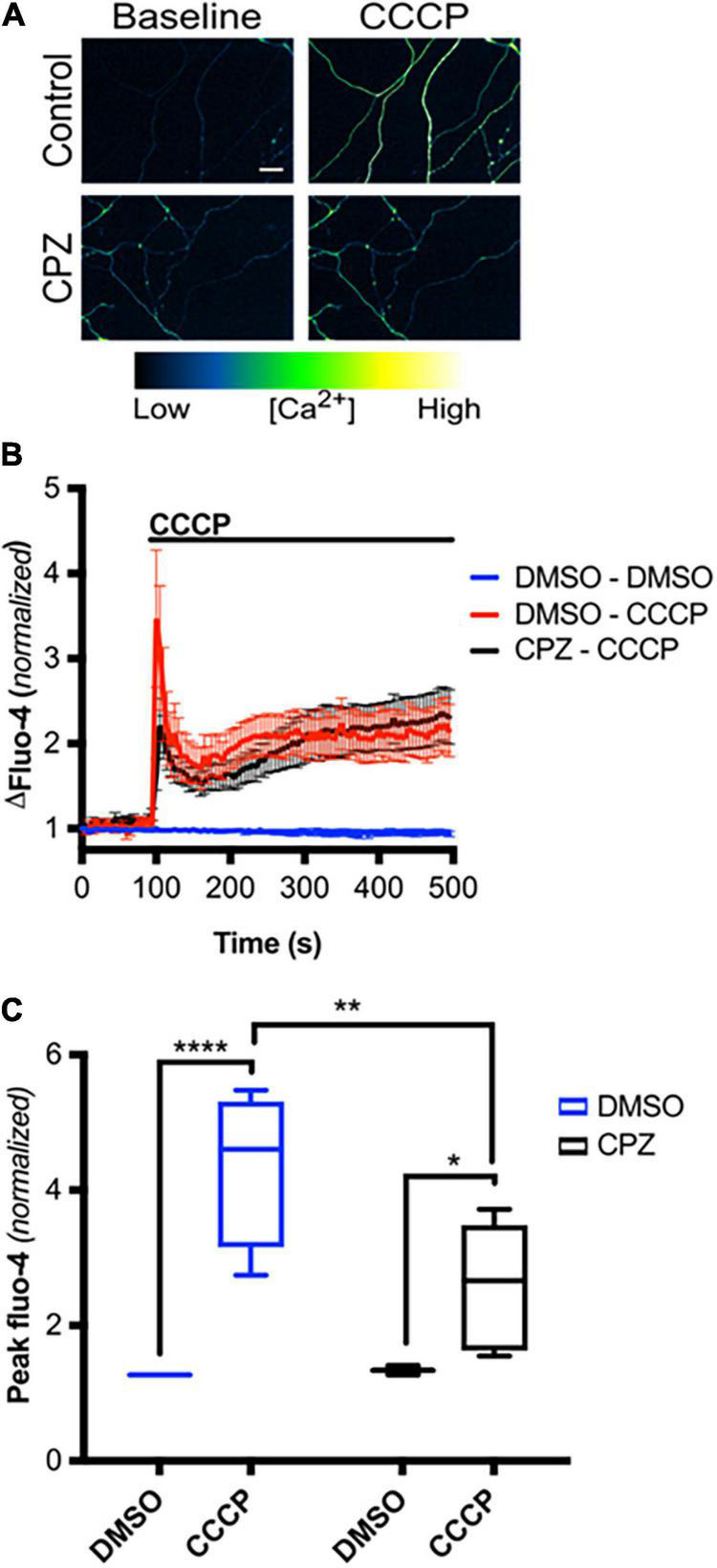
TRPV1 is required for axoplasmic calcium rise following mitochondrial depolarization in intact axons. **(A)** Micrographs of Fluo-4-AM-loaded axons, treated with CPZ (10 μM – 30 min pre-incubation) or DMSO (0.1% v/v – 30 min pre-incubation), before and immediately after CCCP treatment (50 μM) (scale bar indicates 25 μm). **(B)** Changes in Fluo-4 intensity before and after CCCP treatment, normalized to intensities at *t* = 0 s [*n* = 8 (DMSO – DMSO, DMSO – CCCP), 6 (CPZ – CCCP)]. Values are mean ± SEM. **(C)** Peak Fluo-4-AM intensities immediately following CCCP treatment (taken from panel **B**), normalized to intensities at *t* = 0 s. Values are minimum, 1st quartile, median, 3rd quartile, maximum. Statistical significance was assessed using a two-factor ANOVA followed by Tukey’s *post hoc* comparison, *p < 0.05, **p < 0.01, ****p < 0.0001.

**FIGURE 7:**
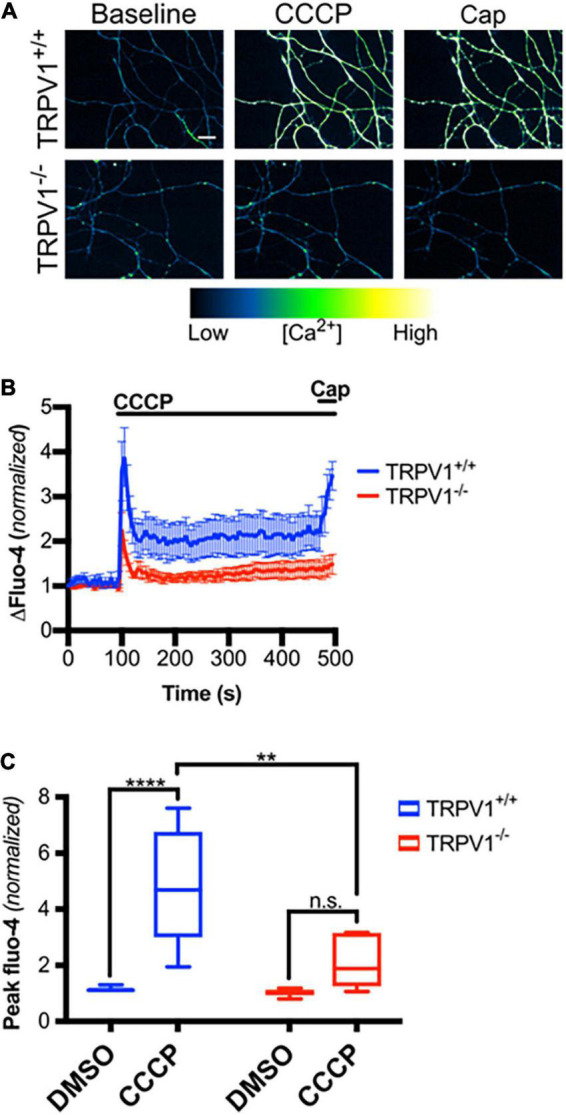
Mitochondrial depolarization in axons causes calcium influx through TRPV1. **(A)** *trpv1*^+/+^ and *trpv1*^– /–^ axons loaded with Fluo-4-AM and treated with CCCP (50 μM; *t* = 100 s) then capsaicin (Cap, 1 μM; *t* = 475 s; scale bar indicates 25 μm). **(B)** Changes in Fluo-4 intensity after CCCP treatment and after Cap treatment, normalized to intensities at *t* = 0 s (*n* = 6 for each group). Values are mean ± SEM. **(C)** Peak Fluo-4-AM intensities immediately following CCCP treatment (taken from panel **B**) normalized to fluorescence intensities at *t* = 0 s for each group. Boxplots are minimum, 1st quartile, median, 3rd quartile, maximum. Statistical significance was assessed using a two-factor ANOVA followed by Tukey’s *post hoc* comparison panel **(C)**, **p < 0.01, ****p < 0.0001.

We next asked if mitochondrial depolarization induced within intact axons can drive TRPV1-mediated Ca^2+^ influx. DRGs were incubated in Ca^2+^-free or NAC-containing media, loaded with Fluo-4 and axons were then imaged in the presence or absence of CCCP a protonophore of the inner mitochondrial membrane (Ly et al., 2003). Figures 5A,B shows that CCCP exposure causes a rapid increase in axonal calcium concentration that stabilizes at a plateau level higher than baseline (peak quantified in Figure 5C). CCCP does not increase intra-axonal Ca^2+^ in neurons maintained in Ca^2+^-free media, indicating that CCCP exposure of intact axons facilitates entry of extracellular calcium. The ROS scavenger NAC strongly attenuates CCCP-induced Ca^2+^ accumulation, suggesting that ROS generation is necessary for the calcium rise induced by mitochondrial depolarization. To determine if TRPV1 was required for CCCP-induced Ca^2+^ influx in DRG axons, we first assessed the effect of the TRPV1 antagonist capsazepine (CPZ). Figure 6 shows that pre-exposure of DRGs to CPZ sharply reduced (by 55%) the calcium peak elicited by CCCP. We then asked if the CCCP-induced Ca^2+^ influx was reduced in *trpv1* null DRG axons; Figures 7A–C shows that Ca^2+^ influx is reduced by 75% in *trpv1*^–/–^ axons.

**FIGURE 8:**
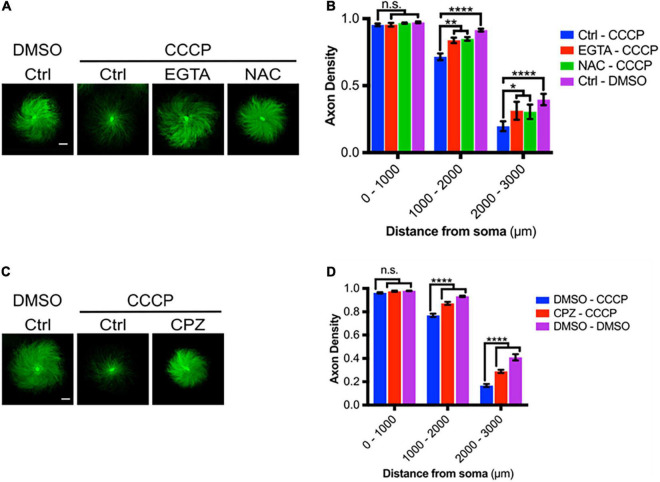
Mitochondrial depolarization leads to axon degeneration. **(A)** DRGs pretreated with either EGTA (6 mM- 30 min pre-incubation) or NAC (20 mM- 30 min pre-incubation), then treated with CCCP (50 μM) and immunostained for β-III tubulin (scale bar indicates 1,000 μm). **(B)** Axon density of DRGs in radial bins of increasing distance from the central soma (*n* = 8 for each group). Values are mean ± SEM. Statistical significance was assessed using a two-factor ANOVA followed by Dunnett’s test relative to relative to CCCP control (Ctrl – CCCP). **(C)** DRGs pretreated with either CPZ (10 μM) or DMSO (0.1% v/v), treated with CCCP (50 μM) and immunostained for β-III tubulin (scale bar indicates 1,000 μm). **(D)** Axon density of DRGs in radial bins of increasing distance from the central soma (*n* = 9 for each group). Values are mean ± SEM. Statistical significance was assessed using a two-factor ANOVA followed by Dunnett’s test relative to DMSO – CCCP. *p < 0.05, **p < 0.01, ****p < 0.0001.

Carbonyl cyanide m-chlorophenyl hydrazine causes rapid degeneration of DRG axons (Figures 8A,B) and we next asked whether this degeneration was promoted by ROS and by TRPV1-mediated Ca^2+^ entry. Figures 8A,B shows that CCCP-mediated degeneration is significantly reduced by ROS scavenging with NAC, by calcium chelation with EGTA, and by TRPV1 blockade using CPZ (Figures 8C,D). Taken together, these results are consistent with the hypothesis that CCCP-dependent ROS production drives TRPV1-dependent Ca^2+^ influx and thereby promotes axon degeneration.

## Discussion

The molecular signaling events that mediate axon destruction during development and after injury have been the subject of intense study for many years. A hallmark event in peripheral nervous system axons subjected to lesion is the rise in intra-axonal calcium, first near the injury site and later throughout the entire distal axon. The later rise in intra-axonal calcium precedes overt degeneration and appears to play a critical role in activating the pathways that ultimately destroy the axon. Early studies established that the late phase of calcium rise relies mainly on the influx of extracellular ions but the specific channels that mediate this influx and mechanisms and activate them remain largely uncharacterized. We recently showed that the cation channel TRPV1 plays a critical role in mediating normal developmental loss of peripheral axons (Johnstone et al., 2019), and here we considered the possibility that TRPV1 may participate in the destructive pathways that result in axon destruction after acute injury. Using pharmacologic and genetic approaches, we show that TRPV1 channels account for a large proportion of the calcium influx required for axon degeneration after axonal lesion and that generation of mitochondrial ROS is required for the Ca^2+^ entry in this setting.

A range of stimuli that include heat, capsaicin, acids and prostaglandins activate TRPV1. Oxidizing agents, including ROS are also well known for their ability to activate TRPV1 or sensitize it to activation by other stimuli. We investigated ROS as a TRPV1 activator after axotomy because ROS accumulates in distal axons after axotomy, and ROS scavengers protect severed axons from degeneration (Welin et al., 2009; Wakatsuki et al., 2015; Frati et al., 2017). Consistent with this, we found that NAC not only prolonged axon integrity after lesion but also sharply reduced Ca^2+^ influx following axotomy.

There are two main potential sources of ROS production after axotomy, NOX complexes and mitochondria. ROS produced by NOX complexes likely make up a relatively small proportion of the total produced after axotomy but nonetheless have been implicated in calcium-independent cytoskeletal degradation (Wakatsuki et al., 2011, 2015). Mitochondria produce large amounts of ROS after axotomy, and the opening of the mitochondrial permeability transition pore allows mitochondrial ROS to enter the axoplasm. Blocking the mitochondrial permeability transition after axotomy protects axons, indicating that ROS derived from mitochondrial may play an essential role in driving axonal destruction (Barrientos et al., 2011; O’Donnell et al., 2013). Here we show that agents that scavenge cytosolic ROS (NAC) or more specifically scavenge mitochondrial ROS (mitoQ) block the late-stage calcium influx that occurs after axotomy, consistent with the hypothesis that mitochondrial ROS are required for TRPV1 activation after axotomy. We also found that NAD^+^ supplementation, which blocks the loss of mitochondrial potential after axotomy, also blocked the late phase of intra-axonal calcium accumulation in severed axons.

These data indicate that mitochondrial ROS generated after axotomy are critical for the TRPV1 activation and Ca^2+^ influx that occurs prior to overt axonal degeneration. The critical role of mitochondrial ROS in this pathway was reinforced by experiments showing that exposure of intact axons to CCCP resulted in a rapid rise in intra-axonal Ca^2+^ that was largely dependent on ROS generation (blocked by NAC) and the subsequent activation of TRPV1 (reduced by 75% in TRPV1 null axons). Interestingly, a recent study established that CCCP-induced mitochondria depolarization and axonal injury is drastically reduced in superior cervical ganglion from *Sarm 1^–/–^* and from the WLD*^s^* mutant. Placing mitochondrial depolarization upstream of SARM1 and NMNAT2 failure during axotomy (Loreto et al., 2020). Although TRPV1 seems to play the predominant role in this setting, it is noteworthy that TRPV1 is only expressed in around 55% of dorsal root ganglion neurons (Han et al., 2016) and that other ROS-sensitive Ca^2+^ channels that are also expressed in peripheral sensory axons, including TRPA1 (Koivisto et al., 2014) and TRPM8 (Takashima et al., 2010; Nocchi et al., 2014) may contribute to these effects. Indeed, the observation that capsazepine almost fully suppresses the calcium increase following axotomy whereas calcium level increases are reduced but not entirely suppressed in axotomized TRPV1^–/–^ axons indicates that CPZ not only targets TRPV1 but also targets related TRP channels in this setting (Liu et al., 2001).

Taken together, these data establish a role for TRPV1-dependent calcium fluxes in the degenerative pathways that are activated by axotomy of peripheral neurons examined *in vitro*. Further studies conducted *in vivo* will be required to characterize the precise role of TRPV1 during axonal injuries under physiological circumstances.

A working model supported by our data and previous works is that axotomy results in the rapid loss of NAD^+^, leading to mitochondrial ROS production and depolarization. Mitochondrial ROS enters the axoplasm *via* the mitochondrial permeability transition pore and targets reactive cysteines on TRPV1 and possibly other channels. These modifications facilitate extracellular Ca^2+^ entry which promotes axonal degeneration through mechanisms that include calpain-mediated cytoskeletal degradation (George et al., 1995; Ma et al., 2013; Yang et al., 2013).

## Data Availability Statement

The original contributions presented in the study are included in the article/supplementary material, further inquiries can be directed to the corresponding authors.

## Ethics Statement

The animal study was reviewed and approved by the UBC Animal Care Committee.

## Author Contributions

BK performed experiments, analyzed the data, produced the figures and wrote the initial draft of the manuscript. AJ performed experiments, analyzed the data, and reviewed the manuscript. JG and PB designed the study, reviewed and analyzed the data, wrote and edited the manuscript. All authors contributed to the article and approved the submitted version.

## Conflict of Interest

The authors declare that the research was conducted in the absence of any commercial or financial relationships that could be construed as a potential conflict of interest.

## Publisher’s Note

All claims expressed in this article are solely those of the authors and do not necessarily represent those of their affiliated organizations, or those of the publisher, the editors and the reviewers. Any product that may be evaluated in this article, or claim that may be made by its manufacturer, is not guaranteed or endorsed by the publisher.
